# Two ischemic stroke events within 48 h: a case report of an unusual presentation of thrombotic thrombocytopenic purpura

**DOI:** 10.1186/s12883-023-03073-1

**Published:** 2023-01-28

**Authors:** Melika Jameie, Sanaz Heydari, Mojdeh Ghabaee, Hamed Amirifard

**Affiliations:** 1 Iranian Center of Neurological Research, Neuroscience Institute, Tehran University of Medical Sciences, Tehran, Iran; 2 Neuroscience Research Center, Iran University of Medical Sciences, Tehran, Iran; 3 Neurology Department, Imam Khomeini Hospital Complex, Tehran University of Medical Sciences, Tehran, Iran

**Keywords:** Purpura, Thrombotic thrombocytopenic, Ischemic stroke, Thrombotic microangiopathies, Anemia, Hemolytic, ADAMTS13 protein, Case report

## Abstract

**Background:**

Thrombotic thrombocytopenic purpura (TTP) considers a rare cause of ischemic stroke (IS). We reported a case of a newly diagnosed patient with acquired immune-mediated TTP (iTTP), in whom two IS events developed during 48 h.

**Case presentation:**

A 59-year-old diabetic male was presented to the hospital 24 h after symptoms onset, including left hemiparesis, dysarthria, and decreased consciousness. A brain CT scan was performed with the suspicion of acute IS, indicating infarct lesions in the right middle cerebral artery (MCA) territory. The patient was not eligible for thrombolytic therapy due to admission delay. Over the next 24 h, the patient’s neurological condition deteriorated, and the second brain CT scan showed new ischemic lesions in the left MCA territory. Initial laboratory evaluation indicated thrombocytopenia without evidence of anemia. However, in the following days, thrombocytopenia progressed, and microangiopathic hemolytic anemia (MAHA) developed. The ADAMTS-13 (a disintegrin and metalloproteinase with a thrombospondin type 1 motif, member 13) activity and inhibitors assay confirmed the diagnosis of iTTP. The patient underwent plasma exchange activity and inhibitors assay confirmed the diagnosis of iTTP. The patient underwent and pulse IV methylprednisolone. Rituximab was also added due to the refractory course of the disease. After a prolonged hospital course, he had considerable neurologic recovery and was discharged.

**Conclusions:**

Clinicians should consider two points. First, TTP should be considered in any patient presenting with IS and having thrombocytopenia or anemia without other symptoms of TTP. Second, worsening the patient's condition during hospitalization may indicate a new stroke and should be investigated immediately.

## Background

Coagulation disorders account for less than 5% of ischemic strokes (IS) [1]. Thrombotic thrombocytopenic purpura (TTP) is a rare, though severe, inherited or acquired disease caused by severe deficiency of the Von Willebrand factor-cleaving serine protease, ADAMTS-13 (a disintegrin and metalloproteinase with a thrombospondin type 1 motif, member 13) [2]. The exact incidence and prevalence figures are not available [3]. However, various numbers have been reported, from 2.2 cases per million population per year to 3.7 [4, 5] or even up to 13 cases per million population, according to a French national registry [6]. Patients with TTP might present with severe thrombotic events such as IS, either initially or during the treatment [7–10]. Stroke develops in 8.16% of patients admitted with TTP, according to the findings from the Nationwide Inpatient Sample (NIS), Healthcare Cost and Utilization Project (HCUP) [11]. Classic clinical manifestations include pentad of fever, microangiopathic hemolytic anemia (MAHA), thrombocytopenic purpura, renal dysfunction, and neurologic symptoms [3, 12]. Laboratory findings include thrombocytopenia and evidence of MAHA, including schistocytes, reticulocytosis, indirect hyperbilirubinemia, undetectable haptoglobin, and high lactate dehydrogenase (LDH) [12]. The diagnosis is suspected based on the clinical and laboratory findings and confirmed by very low ADAMTS-13 levels [12]. Of note, in the absence of other obvious causes, evidence of MAHA or thrombocytopenia without the presence of the classic pentad is strongly suggestive of TTP, hence the importance of immediate total plasma exchange (TPE) [13].

We reported a case of a newly diagnosed patient with acquired immune-mediated TTP (iTTP), in whom two IS events developed during a period of 48 h. This circumstance is unique in several ways. To begin with, iTTP is a rare disorder. Second, stroke is considered to be an unusual inaugural presentation of TTP. Third, there are currently very few reports of new ischemic lesions during hospitalization in individuals with iTTP following the first ischemic stroke event [14, 15]. Notably, some of these patients had the congenital type of the disease, making our patient even more intriguing.

## Case presentation

A 59-year-old diabetic male was referred to our emergency room following left-sided weakness, hemifacial paresis, difficulty speaking, and drowsiness from one night before (National Institute of Health Stroke Score (NIHSS) at admission = 10). There was no history of recent head trauma. Family history of premature stroke or thrombotic events was also negative. The patient was a non-smoker, albeit an opiate user. The initial physical exam showed an elderly, confused male who could not answer the questions or follow commands cooperatively. Vitals were as follow: T = 37.4 °C, BP = 150/90 mmHG, HR = 73 bpm (beats per minute), RR = 16/minute, and O_2_ saturation in the room air = 97%. An electrocardiogram (ECG) showed normal sinus rhythm. On initial physical examination, no signs of pallor, jaundice, petechiae, or organomegaly were evident. Except for left facial paresis, cranial nerves examination was normal. Motor examination revealed left hemiparesis, with a force of 3/5 in the left upper extremity and 4/5 in the left lower extremity. The grimace to pinprick was reduced on the left side. The plantar reflex showed upward movement in the left side (Babinski sign).

### Investigations and treatment

**Fig. 1:**
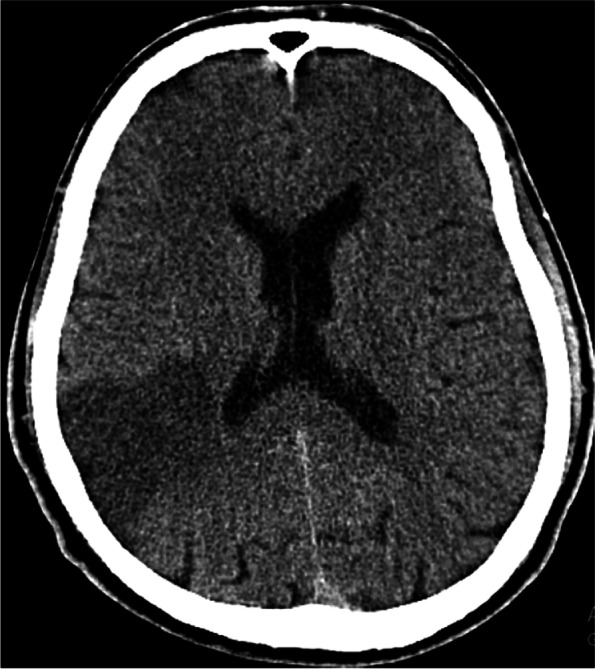
Brain CT scan on admission showed the right parietotemporal lobe infarct

**Fig. 2:**
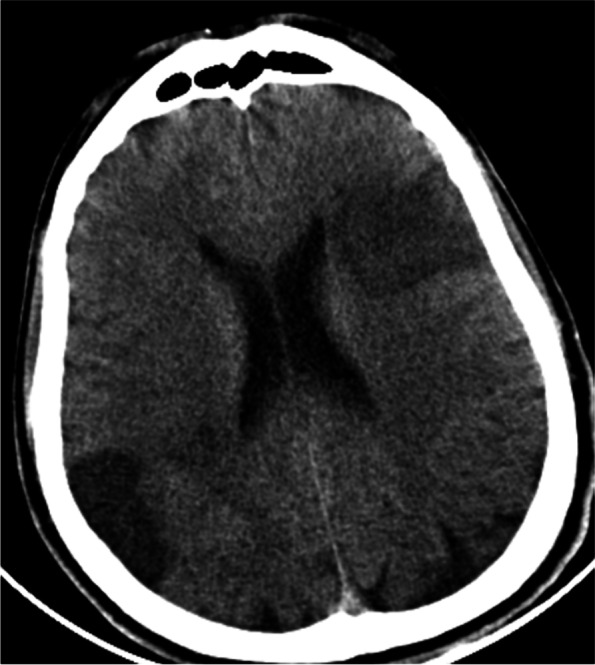
Brain CT scan 24 h after admission showed a new infarct in the left frontal lobe

The patient underwent a brain CT scan with suspicion of IS. Of note, since the patient's symptoms had been initiated more than 24 h ago, he was not eligible to receive intravenous thrombolysis (IVT) or thrombectomy [16]. The first brain CT scan showed an acute stroke in the right middle cerebral artery (MCA) territory, including the right parietotemporal lobe infarct (Fig. 1). The patient's level of consciousness deteriorated over the next 24 h, necessitating further evaluation; the second brain CT scan revealed evidence of new ischemic lesions in the left MCA territory, including the left frontal lobe (Fig. 2). Progressive thrombocytopenia was found in the patient (initial platelet count: 77*${10}^{3/}\mathrm{\mu L}$ reduced to 21*${10}^{3/}\mathrm{\mu L}$). Although the patient was not anemic at first (initial Hgb: 15 g/dL), evidence of hemolytic anemia gradually emerged, including a reticulocyte percentage of 2.6%, reduced Hgb (from 15 g/dL to 11.8 g/dL on day 6), indirect hyperbilirubinemia (total bilirubin 1.9 mg/dL increased up to 4.4 mg/dL, and indirect bilirubin 1.1 mg/dL increased up to 3.1 mg/dL), and elevated LDH (1148 U/L). Additionally, acute phase reactants, including ESR, CRP, ferritin, fibrinogen, and D-dimer, were also elevated. Troponin, coagulation tests, liver function tests, lipid profile, and electrolytes were normal. Peripheral blood smear (PBS) showed occasional schistocytes. Direct and indirect Coombs tests were negative. TTP diagnosis was highly suspected based on the patient’s clinical manifestations and progressive thrombocytopenia. Therefore further evaluation was performed, indicating an ADAMTS-13 activity level of 0.3 IU/mL (our laboratory cut-off for a positive value was < 0.4 IU/mL) and ADAMTS-13 inhibition screen of 18 U/mL (our laboratory cut-off for a positive value was > 15 U/mL), confirming the diagnosis of iTTP. Other Serologic evaluations (antinuclear antibody (ANA), anti-double strands DNA antibody (anti-dsDNA Ab), perinuclear anti-neutrophil cytoplasmic antibodies (P-ANCA), cytoplasmic anti-neutrophil cytoplasmic autoantibody (C-ANCA), rheumatoid factor (RF), complement 3 (C_3_), complement 4 (C_4_), anticardiolipin IgM and IgG antibodies, antiphospholipid IgM and IgG antibodies, and anti–Sjögren's-syndrome-related antigen A and B autoantibodies (SSA and SSB) were within normal limits. HIV, hepatitis B virus, and hepatitis C virus serology were also nonreactive (Table 1).

**Table 1:** The results of patients' laboratory evaluation at presentation, day 7 hospitalization, and discharge

Laboratory test (unit)	Initial value	Day 7 hospitalization	Discharge value
WBC (${10}^{3/}\mathrm{\mu L}$)	10.8		8
Hgb (g/dL)	15	12.3	12
MCV (fL)	86.5		
MCH (pg)	30.9		
Reticulocyte (%)	3.2	4	
Corrected reticulocyte (%)	1.7	2.6	
PLT (${10}^{3/}\mathrm{\mu L}$)	77	25	189
PT (sec)	13.1		13.1
PTT (sec)	28		26
INR	1.0		1.0
Glucose (mg/dL)	268		
HbA1c (%)	> 14	12.3	
BUN (mg/dL)	18		23
Cr (mg/dL)	0.9		0.7
Total Bilirubin (mg/dL)	1.9	4.4	1.4
Direct Bilirubin (mg/dL)	0.8	1.3	0.4
LDH (U/L)	1148		456
ESR (mm/h)	49		
CRP (mg/l)	16		
D-Dimer (µg/l)	1932		
Fibrinogen (mg/dL)	609		
Ferritin (ng/mL)	910		

*WBC* White blood cells, *Hgb* hemoglobin, *MCV* Mean corpuscular volume, *MCH* Mean corpuscular hemoglobin, *PLT* Platelet, *PT* Prothrombin time, *PTT* Partial thromboplastin time, INR International normalized ratio, *HbA1c* Glycosylated hemoglobin, *BUN* Blood urea nitrogen, *Cr* Creatinine, *LDH* Lactate dehydrogenase, *ESR* Erythrocyte sedimentation rate, *CRP*, C reactive protein

**Fig. 3:**
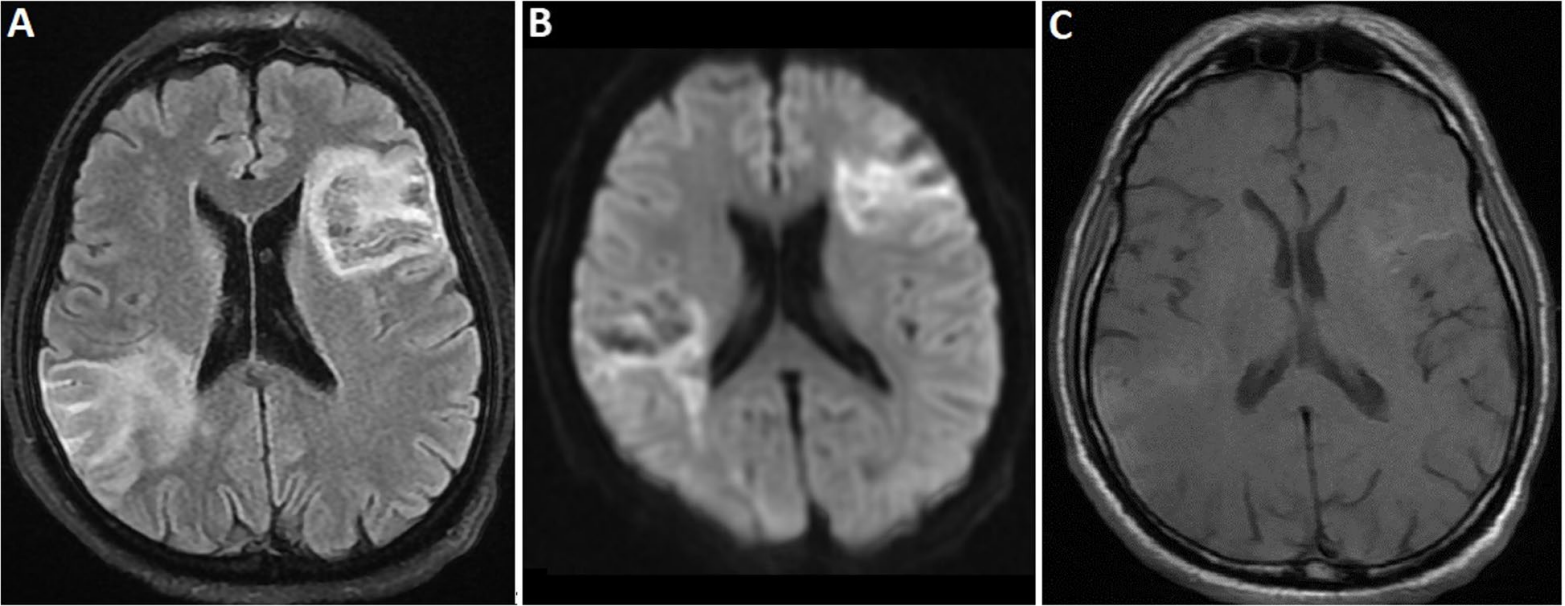
Brain MRI on day 8 showed subacute infarctions with restriction and mild hemorrhagic transformation in the right parietotemporal, left frontal lobe, and left centrum semiovale; A: Diffusion-weighted imaging (DWI); B: Fluid attenuated inversion recovery (FLAIR); C: T1-weighted

Although the cardioembolic source of stroke was suspected due to the bihemispheric infarcts, ECG Holter monitoring, trans-thoracic, and trans-esophageal echocardiography revealed no signs of arrhythmia, clot, or vegetation, and the ejection fraction (EF) was 50–55%. Brain MRI on day 8 demonstrated evidence in favor of subacute infarctions with restriction and mild hemorrhagic transformation in the right parietotemporal, left frontal lobe, and left centrum semiovale (Fig. 3). There was no evidence of venous thrombosis or vascular lesions on magnetic resonance angiography (MRA) and magnetic resonance venography (MRV). Since malignant bone marrow infiltration is one of the main differential diagnoses in a patient with MAHA and thrombocytopenia [14], hematology was consulted. They asked for a bone marrow aspiration and biopsy (day 8), which showed normocellular marrow with increased erythroid series. Informed consent was obtained, and the patient underwent therapeutic plasma exchange (TPE) on day 8 with iTTP diagnosis. Consequently, the patient’s level of consciousness improved so that he was completely alert and responsive 9 days after TPE (day 17 hospitalization). The platelet count also gradually increased from 21*${10}^{3/}\mathrm{\mu L}$ up to 91*${10}^{3/}\mathrm{\mu L}$ a week after TPE initiation, although it again started to decrease. Hence, pulse IV methylprednisolone therapy was added on day 17 of hospitalization for 3 days, followed by oral prednisolone (1 mg/kg). Rituximab (750 mg/m^2^) was also initiated on day 30 of hospitalization. The patient's clinical condition, as well as the MAHA, gradually improved (NIHSS at discharge = 3), and the PLT count reached 189*${10}^{3/}\mathrm{\mu L}$ at discharge (48 days of hospitalization, including 17 days at the stroke care unit (SCU), 5 days at the neurology ward, and 26 days at the hematology ward). During the follow-up, the neurological symptoms progressively improved, and he did not experience further relapses or report adverse effects. (modified Rankin Scale = 1, 75 days after discharge). The patient’s timeline is shown in Table 2.

**Table 2:** An overview of the patient’s timeline during hospitalization

Day 0	Initiation of stroke-related symptoms
Day 1	Admission to the hospital
	The first brain CT showed the right parietotemporal lobe infarct
	Detection of thrombocytopenia
Day 2	Worsening of patient’s neurological condition
	The second brain CT showed a new infarct in the left frontal lobe
Day 6	Decrease in hemoglobin ≤ 13 g/dL
	Evidence of MAHA
Day 7	ADAMTS-13 activity and inhibitors assay
Day 8	Brain MRI showed subacute infarctions with restriction and mild hemorrhagic transformation in the right parietotemporal, left frontal lobe, and left centrum semiovale
	Bone marrow aspiration and biopsy
	Initiation of therapeutic plasma exchange
Day 15	Platelet count reached 91*${10}^{3/}\mathrm{\mu L}$
Day 17	Considerable improvement neurologically
	Platelet count started to decrease again
	Initiation of pulse IV methylprednisolone
Day 20	Initiation of oral prednisolone
Day 30	Rituximab was added
Day 48	Discharged

*MAHA* Microangiopathic hemolytic anemia, *ADAMTS-13* A disintegrin and metalloproteinase with a thrombospondin type 1 motif member 13, *IV* Intravenous

## Discussion

In this study, we reported a 59-year-old male presented with hemiparesis, dysarthria, and decreased consciousness who developed two ischemic stroke events during 48 h, which is extremely rare. The results of neuroimaging revealed evidence of infarction in the right (at admission) and left (24 h after admission) MCA territories, respectively. Initial laboratory findings indicated progressive thrombocytopenia. However, MAHA was not found at presentation. Further evaluations, including measurement of ADAMTS-13 activity level and ADAMTS-13 inhibitor, confirmed the diagnosis of iTTP. The patient underwent therapeutic plasma exchange and pulse IV methylprednisolone therapy. Rituximab was also given since it could reduce relapses, and is increasingly recommended for use in refractory cases [17, 18]. Notably, studies showed that although iTTP considers an autoimmune disease, ADAMTS-13 inhibitors are not easily detectable in many patients [19], which was not the case in our patient. Similar to the prolonged course of treatment in our patient, studies showed that high titers of inhibitors of ADAMTS-13 are associated with delayed response to therapy, high risk of complications, and higher rates of refractory disease and relapses [19, 20]. Of note, while there are reports of patients with TTP who presented with IS as an atypical presentation [15, 21], there are very few reports of new ischemic lesions during hospitalization following the first ischemic stroke event [14, 15]. One was a 70-year-old female who presented with confusion, fever, and gastrointestinal symptoms who deteriorated neurologically during hospitalization, and the brain MRI revealed multiple acute and subacute infarcts in both cerebral hemispheres, midbrain, and right cerebellum. The patient had a history of hypertension, diabetes, rheumatoid arthritis, two miscarriages complicated by disseminated intravascular coagulation, two transient ischemic attacks, and a left occipital infarct and she was diagnosed with congenital TTP [14]. Another one was a 43-year-old female who was admitted with left hemiparesis, homonymous hemianopia, and dysarthria. The first brain CT showed an acute stroke in the right MCA territory, and follow-up MRIs during the first week of her hospitalization demonstrated new ischemic lesions in various territories [15].

TTP is a relapsing and life-threatening condition characterized by clot formation in small blood vessels [22]. Clinical manifestations vary from mild nonspecific symptoms to severe organ damages, including kidney and brain, and death [3]. Neurologic involvements, such as headaches, confusion, focal neurologic deficit, etc., are common as the initial presentation of TTP usually as a result of diffuse microthrombi formation in the microvasculature of the central nervous system (CNS), resulting in transient or permanent ischemic brain damage [3, 7–9, 23]. However, TTP considers a rare cause of ischemic stroke [15], although there are similar cases in the literature of patients being diagnosed with TTP after a cerebrovascular accident [9, 21, 24–26]. A recent study on patients with TTP who presented with neurological symptoms suggested that old age, hypertension, smoking, and high plasma concentrations of anti-ADAMTS-13 IgG may be the risk factors for developing cerebral infarction in these patients [27]. Furthermore, they reported no association between iTTP stroke and diabetes, however, our patient was diabetic [27]. Additionally, it is suggested that decreased level of ADAMTS-13 is associated with stroke recurrence after remission [28], hence the importance of patients’ follow-up.

A literature review conducted in 2018 reported 17 cases of IS due to iTTP [15]. Interestingly, none of the patients showed the classical TTP pentad, and wide non-specific heterogeneity in clinical manifestations was observed [15]. The combination of thrombocytopenia and hemolysis was found in less than half of the patients (41%). In 41% of cases, the stroke was multifocal and included major artery strokes, with proximal occlusion in 3 cases. Considerably, refractory and relapsing forms were observed in nearly half of the patients (47%). They reported small and large artery strokes in these patients, with the majority being multifocal and without a specific pattern [15]. These findings confirm the claim that although TTP is classified as a thrombotic microangiopathic condition, which results in small vessels obstruction, it should also be considered in patients with proximal intracranial artery occlusion [9, 15, 24, 29].

## Conclusion

In alignment with previous studies, this study showed that although TTP is considered an uncommon cause of ischemic stroke, it should be considered in every IS patient with isolated anemia or thrombocytopenia even in the absence of other classic symptoms or laboratory findings. This study also suggests that clinicians should consider the possibility that worsening of the patient’s clinical condition could be attributed to emerging of new infarcts, emphasizing the importance of early diagnosis and treatment of iTTP to prevent multiple infarcts and a poor prognosis.

## Data Availability

Data sharing is not applicable to this article as no datasets were generated or analyzed during the current study.

## Abbreviations

ADAMTS-13: A disintegrin and metalloproteinase with a thrombospondin type 1 motif, member 13
ANA: Antinuclear antibody
Anti-dsDNA: Anti-double strands DNA
Bpm: Beats per minute
C-ANCA: Cytoplasmic antineutrophil cytoplasmic autoantibody
CNS: Central nervous system
C3: Complement 3
C4: Complement 4
ECG: Electrocardiogram
EF: Ejection fraction
HCUP: Healthcare cost and utilization project
Hgb: Hemoglobin
IS: Ischemic stroke
iTTP: Immune-mediated thrombotic thrombocytopenic purpura
IVT: Intravenous thrombolysis
LDH: Lactate dehydrogenase
MAHA: Microangiopathic hemolytic anemia
MCA: Middle cerebral artery
MRA: Magnetic resonance angiography
MRV: Magnetic resonance venography
NIHSS: National institute of health stroke score
NIS: Nationwide inpatient sample
P-ANCA: Perinuclear anti-neutrophil cytoplasmic antibodies
PBS: Peripheral blood smear
RF: Rheumatoid factor
SSA: Sjögren's-syndrome-related antigen A
SSB: Sjögren's-syndrome-related antigen B
TPE: Therapeutic plasma exchange
TTP: Thrombotic thrombocytopenic purpura
